# Molecular and statistical weaknesses of the p-tau217/Aβ_1−42_ plasma ratio for alzheimer’s diagnosis

**DOI:** 10.1007/s00109-026-02676-8

**Published:** 2026-05-12

**Authors:** Christian Griñán Ferré, Mercè Pallàs, Rafael Franco

**Affiliations:** 1 Department of Pharmacology and Medicinal Chemistry, School of Pharmacy and Food Sciences, University of Barcelona, Barcelona, Spain; 2 Institut de Neurociències de la Universitat de Barcelona, Barcelona, Spain; 3 Centro de Investigación en Red, Enfermedades Neurodegenerativas (CIBERNED), Instituto de Salud Carlos III, Madrid, Spain; 4 Department of Biochemistry and Molecular Biomedicine, Universitat de Barcelona, Barcelona, Spain; 5 Institute of Theoretical and Computational Chemistry (IQTC, Barcelona University, Universitat de Barcelona, Barcelona, Spain

**Keywords:** Alzheimer’s disease biomarkers, Plasma p-tau217, Diagnostic accuracy, Blood-brain barrier permeability, Circular validation

## Abstract

The FDA’s approval of the Lumipulse G p-tau217/Aβ_1−42_ plasma ratio enhances access to Alzheimer’s diagnostics but risks confusing convenience with biological accuracy. The assay is scalable and non-invasive, yet it relies on a ratio of two markers that are unstable and only partly specific to the disease, raising concerns about reproducibility and interpretation. The reported performance is solid in carefully selected groups, but is likely to be less robust in broader real-world populations with lower disease prevalence, mixed pathologies, and higher comorbidity. If biomarker-based enrollment is shaped by imperfect specificity, misclassification may propagate into trial recruitment, treatment-effect estimates, and downstream validation. This concern is amplified when biomarkers are validated within partially circular frameworks in which plasma assays, positron emission tomography, cerebrospinal fluid markers, and clinical diagnosis reinforce one another without fully independent neuropathological confirmation. Blood-based assays remain promising, but their clinical use should be guided by rigorous analytical scrutiny, broad validation across diverse populations, standardized pre-analytical handling, and transparent data sharing. The aim of biomarker science should be not striking receiver operating characteristic curves in curated cohorts, but biological fidelity across human heterogeneity and validation grounded in mechanism. Until then, the p-tau217/Aβ_1−42_ ratio is best regarded as a useful contextual or research tool rather than a standalone diagnostic benchmark, so that precision medicine does not rest on associations whose causal and mechanistic basis remains insufficiently established. Its most appropriate use may be in longitudinal monitoring within the same individual, where changes over time may be more informative than a single threshold-based diagnostic result.

## Introduction

The FDA has cleared the Lumipulse G ptau217/β-Amyloid_1−42_ plasma ratio as the first blood test to help evaluate Alzheimer’s disease (AD) in symptomatic adults aged 55 and older, i.e. the assay should not be interpreted as a definitive diagnostic tool. Its clearance has been presented as an important step toward more accessible AD assessment, particularly as early-stage treatments such as lecanemab and donanemab enter clinical use and timely evaluation becomes increasingly relevant. The promise of this approach is clear, but so are its limitations. From a statistical perspective, false positives or denominator-driven shifts in the ratio could affect patient stratification, trial recruitment, and the interpretation of treatment effects. As adoption grows, these limitations will require careful consideration if diagnosis and treatment pathways are to remain appropriately grounded.

Importantly, FDA clearance was based largely on performance in enriched cohorts, in which participants were selected on the basis of cognitive symptoms and were often pre-screened with imaging biomarkers [1, 2]. The positive predictive value (PPV) in broader populations remains uncertain and varies substantially with disease prevalence. At a 10% prevalence, typical of some memory-clinic settings, even a test with 90% sensitivity and 90% specificity yields a PPV of only 50%, meaning that half of positive results could be false positives [3, 4]. In primary care, where AD prevalence may be closer to 1–5%, the PPV falls further, to roughly 8–32%, making the test unsuitable for screening.

## Fundamentals of clinical chemistry

Clinical Chemistry has long relied on blood-based measurements to detect systemic disease, monitor progression, and guide treatment. This paradigm has worked especially well for acute disorders, metabolic diseases, and chronic conditions in which the circulating analyte is chemically well defined and closely linked to pathophysiology. Neurodegenerative diseases remain more difficult because the relevant proteins exist in multiple molecular forms and their peripheral measurement is strongly shaped by pre-analytical and matrix effects.

For chronic diseases, blood-based tests are essential for confirming diagnoses, assessing risk, and monitoring treatment response. In type 2 diabetes, for example, diagnosis and follow-up rely on plasma glucose and glycated hemoglobin (HbA1c), which reflects glycemic control over approximately 8 to 12 weeks. Despite substantial progress, translating brain pathology into reliable peripheral biomarkers for neurodegenerative diseases has proved difficult. Research has focused on aggregation-prone proteins such as α-synuclein in Parkinson’s disease and amyloid-β (Aβ) and phosphorylated tau in AD. Yet α-synuclein, Aβ, and p-tau all exist in multiple molecular forms, with different stabilities, solubilities, and biological relevance.

Even ultrasensitive immunoassays measure composite signals from species whose pathogenic correspondence is only partly defined, while pre-analytical variables such as hemolysis, platelet contamination, and protein degradation introduce substantial variability (Table 1). Pre-analytical handling affects both p-tau217 and Aβ_1−42_ measurements. Hemolysis can increase p-tau217 and decrease Aβ_1−42_, whereas platelet contamination has a high to very high impact on both analytes [5–7]. Time to centrifugation (ideally < 2 h), storage temperature (critical at -80 °C), and repeated freeze-thaw cycles (> 3 cycles) also introduce systematic variability that may approach or exceed biological differences between diagnostic groups [8, 9]. EDTA tubes are generally preferred over other anticoagulants, yet even under standardized protocols the coefficient of variation for Aβ_1−42_ can reach 15–25% [10–12]. The restricted permeability of the blood-brain barrier further weakens the relation between central pathology and peripheral measurement. Accordingly, although the p-tau217/Aβ_1−42_ ratio may have diagnostic utility, it should be adopted cautiously and not treated as a definitive standard until its mechanistic and pathological specificity are better established. The following sections outline the molecular and analytical reasons for that caution.

**Table 1:** Pre-analytical variables affecting plasma biomarker stability

Pre-analytical Variable	Effect on *p*-tau217	Effect on Aβ42	Mitigation Strategy	References
Hemolysis	Variable increase	Significant decrease	Careful phlebotomy	[5, 9]
Platelet contamination	High impact	Very high impact	Standardized centrifugation	[5, 7]
Time to centrifugation	Moderate impact	High impact	< 2 h recommended	[7, 9]
Storage temperature	Critical	Critical	-80 °C storage	[8, 9]
Freeze-thaw cycles	Significant degradation	High degradation	Minimize to < 3 cycles	[9, 12]

### Biochemical structure of ptau217 in blood

Phosphorylation at threonine 217 (T217), located within the proline-rich P1 domain of tau [13–15], has emerged as a candidate biomarker for AD. Even so, current evidence suggests that this site is not disease-specific. The longest tau isoform (2N4R, UniProtKB P10636-8) contains approximately 50 phosphorylation sites in tau isolated postmortem from AD brain tissue. Across all isoforms, mass-spectrometry studies have identified at least 85 distinct phosphorylation sites, with T217 consistently mapped to the P1 region [13, 14, 16].

Tau containing p-tau217 is detected not only in individuals with AD but also in cognitively normal subjects [17, 18]. Although the abundance and co-occurrence of other phosphosites may differ between these groups, phosphorylation at T217 is not confined to disease states. Both the P1 and P2 proline-rich domains undergo extensive phosphorylation, and proteomic analyses show that many of these phosphosites are shared across AD and control samples [17, 18].

This overlap does not merely reflect a technical obstacle, but a fundamental property of tau biology: tau is subject to extensive and heterogeneous phosphorylation, the regulation of which remains incompletely understood. As Wegmann et al. (2021) note: “*the phosphorylation patterns of physiological and pathological tau are surprisingly similar and heterogeneous*,* making it difficult to identify specific modifications as therapeutic targets and biomarkers for AD”*. The picture is further complicated by tau proteolysis. In plasma, tau is present as a mixture of full-length and truncated fragments, each with distinct phosphorylation patterns and potentially different functional or diagnostic relevance [17].

Because p-tau217 is detectable in cognitively normal individuals, greater disease specificity at the plasma level will likely require molecular context beyond the isolated T217 phospho-epitope. Relevant contextual dimensions may include the co-occurrence of other tau phosphosites, such as p-tau181 or p-tau231, the distinction between full-length tau and truncated fragments, information on fragment origin and proteolytic processing, and orthogonal markers reflecting amyloid biology, neurodegeneration, glial activation, or blood-brain barrier dysfunction. In that sense, specificity is more likely to emerge from a structured molecular profile or multimarker signature than from any single plasma phospho-signal taken in isolation.

A possible future strategy to partially mitigate this limitation would be to combine immunocapture of pT217-containing tau species with targeted mass spectrometry. Such an approach would not eliminate the underlying heterogeneity of plasma tau, but it could help resolve part of the composite signal by distinguishing selected truncated fragments and multiphosphorylated proteoforms rather than collapsing them into a single epitope-based measurement. In that sense, future assay design may become more informative by being fragment-aware and proteoform-aware, rather than by de facto assuming that plasma p-tau217 reflects a single molecular entity.

### Is composite metric using Aß_1−42_ the answer?

Repeated measurement of p-tau217 in an individual may be useful for tracking the rate of disease progression [19, 20], but its heterogeneity limits its value for initial diagnosis or baseline patient stratification. N-terminal and mid-region tau fragments that retain the T217 epitope show distinct profiles in AD versus controls, yet current immunoassays cannot distinguish full-length pT217-tau from pT217-containing fragments and therefore yield a composite signal of uncertain composition [21]. Calpain-mediated and caspase-mediated cleavage further generate fragments with different stability profiles and blood-brain barrier permeability, complicating peripheral measurement still further [22, 23].

At present plasma p-tau217 should be understood as a heterogeneous composite signal rather than a single molecular species, which limits its mechanistic specificity in cross-sectional diagnosis. As normalization is often used to improve comparability in complex biological systems, the relevant question, therefore is whether Aβ_1−42_ can serve as a biochemically and analytically appropriate denominator in a derived index that supports accurate diagnosis.

Dividing plasma p-tau217 by Aβ_1−42_ is one approach to addressing the fact that p-tau217 in blood does not represent a single molecule, but rather the aggregate signal of multiple tau species that share phosphorylation at Thr217. The ratio may be clinically useful in specific settings because it compresses two signals into a convenient composite measure. That utility, however, is conditional rather than universal: the ratio does not correct the underlying molecular heterogeneity, nor does it convert plasma p-tau217 into a universally stable biomarker.

In blood, Aβ_1− 42_ is an intrinsically disordered peptide that rapidly binds to carrier proteins such as albumin and lipoproteins. This binding can mask epitopes, affect detectability, and make the apparent concentration highly dependent on assay format and pre-analytical handling. These technical limitations would explain, beyond differences due to ethnicity, why plasma levels of Aβ_1− 42_ have been reported to range between 2.72 and 11.09 pg/mL (using Single Molecule Array -SIMOA^®^-) or 8.12-29.00 pg/mL (using Innogenetics ELISA) [24, 25].

In addition, a substantial fraction of circulating Aβ is produced peripherally, with platelets representing a major source; they contain more than 90% of blood APP and release Aβ upon activation. Platelet APP processing through β-secretase and γ-secretase generates Aβ_1−40_ and Aβ_1−42_ independently of CNS pathology [5, 26]. The contribution of brain-derived Aβ_1−42_ to plasma levels remains debated, with estimates ranging from less than 10% to about 30%, depending in part on blood-brain barrier integrity [27–29]. Plasma Aβ_1−42_ therefore appears to reflect predominantly peripheral production, with only a variable CNS contribution, which weakens its linkage to brain amyloid burden [5, 26, 30].

Analytical validation of the Lumipulse G assay reports within-run coefficients of variation (CVs) of 2.2–4.1% for p-tau217 and 2.8–5.3% for Aβ_1−42_, whereas between-run and between-laboratory CVs reach 8–12% and 12–18%, respectively. Standard error-propagation theory indicates that the CV of a ratio is approximately the square root of the sum of the squared CVs of the numerator and denominator. Thus, a ratio formed from two measurements that each have a 10% CV would itself have a CV of about 14% [31, 32]. This is a hypothetical illustration intended to clarify the measurement principle, not to suggest that every cohort or platform necessarily exhibits the same degree of compounded uncertainty. Even so, the concern is not rhetorical: under the simplifying assumption of independent errors, it follows directly from standard propagation-of-uncertainty theory for quotients. In a realistic measurement setting, this compounded variability may approach or even exceed the biological effect sizes expected in early-stage disease, thereby increasing the likelihood of false-positive diagnoses among cognitively normal or otherwise healthy individuals.

From an analytical standpoint, the two components of the ratio are not equivalent measurements. Aβ_1−42_ immunoassays generally target the peptide’s intact N- and C-termini and often require detergents to dissociate it from carrier proteins and expose the relevant epitopes. By contrast, p-tau217 assays detect a phospho-epitope present across multiple full-length and truncated tau fragments. A further complication is that neither p-tau217 nor Aβ_1−42_ measures are assay-invariant. Their measured values depend on platform design, antibody specificity, calibrators, and pre-analytical handling, making absolute concentrations and cutoffs non-interchangeable across methods. The FDA clearance of the Lumipulse implementation should therefore be read as validation of one platform under defined conditions, not as evidence that the p-tau217/Aβ_1−42_ quotient is analytically interchangeable across assays or canonically established for diagnosis.

Aβ_1−42_ may improve discrimination in specific platform-bound settings, but it does not constitute a biologically stable denominator that converts plasma p-tau217 into a general-purpose diagnostic metric.

### Further limitations of using ratios in clinical chemistry

In Clinical Chemistry, ratios require explicit validation because they combine two biological quantities, two measurement processes, and two error structures. Their interpretability depends not only on dimensional consistency but also on the chemical identity, stability, and measurement validity of both terms. Unit cancellation alone does not guarantee biological meaning, especially when numerator and denominator differ in matrix behavior, molecular definition, or assay dependence. If the measurement model changes for either analyte, the apparent simplicity disappears. In real-world settings, the denominator often carries biological and pre-analytical behavior unrelated to the numerator, including differences in molecular stability, sample-matrix interactions, or epitope accessibility.

From a foundational perspective, a quotient such as [A]/[B] is interpretable only if both terms represent well-defined and commensurate quantities. Suppose that A and B each denote the absolute concentrations of a single, chemically defined species expressed on the same scale, such as molarity. Under those conditions, the units may cancel mathematically. Even then, however, the meaning of the resulting ratio still depends on the chemical identity, stability, and measurement properties of both measurands.

Mass-based ratios introduce an additional layer of complexity. The Lumipulse assay, for example, handles p-tau217/Aβ_1−42_ ratio as a pg/pg quotient. For between-sample comparisons, this would be defensible if each analyte denotes a single, assay-invariant, chemically well-defined molecular species with constant molar mass across samples. Under those conditions, the mass ratio is proportional to the molar ratio, so both convey the same ordering and relative differences, and the choice between mass and molar units mainly changes the numerical scale. That equivalence disappears, however, as soon as either analyte represents multiple species or exhibits variable composition, for example because of isoforms, post-translational modifications, heterogeneous lipid chains, or complex formation. In that situation, the effective molar mass is no longer fixed, and mass and molar ratios can no longer be interconverted by a single constant.

Because the plasma p-tau217 signal consists of multiple tau fragments, a pg/pg p-tau217/Aβ_1−42_ ratio cannot be accurately translated into a molar ratio, nor can it be regarded as a well-defined normalized measurement. The mass concentration of this epitope-defined mixture does not map directly onto molecule number, so apparent algebraic cancellation of units does not by itself confer biological interpretability.

Denominator drift is further amplified by the broad 95% reference interval observed even in healthy populations [24, 25, 33], much of which appears to reflect methodological and inter-laboratory variability. Added to this, differences in binding partners, clearance routes, and compartmental origins may displace numerator and denominator in opposite directions under common clinical conditions, further undermining the stability of the metric.

### The lack of open data further impacts on analysis of the potential of ptau217/Aß_1−42_ ratio in clinical chemistry

Inter-sample variability, a common feature of human biological samples, necessitates the exploration of analytical strategies that improve the interpretability of measured parameters. Quotient-based normalization using the least variable compound as a reference may not adequately reduce this dispersion, whereas multivariate analysis can be more effective in revealing underlying structure [34].

Irrespective of the analytical platform, p-tau217 and Aβ_1−42_ measurements in plasma are intrinsically paired within each sample, and analyses that preserve that joint structure may be more informative than reducing both analytes to a single quotient.

A statistically appropriate alternative would therefore be to analyze p-tau217 and Aβ_1–42_ jointly as separate but correlated variables rather than collapsing them into a quotient, thereby preserving analytical and biological information that is lost when numerator and denominator are reduced to a single composite.

A further complication is denominator instability in real-world cohorts. Plasma Aβ_1− 42_ values can vary substantially even within the same clinical category. In the above-mentioned studies, among control individuals, the ratio between the maximum and minimum values ranged from 3.6-fold (2.72–11.09 pg/mL) to 4.4-fold (8.12–29.00 pg/mL), depending on the cohort [24, 25]. Variability was also notable among patients with AD in the Biomarker of AmyLoid pepTide and AlZheimer’s diseAse Risk multicenter prospective (BALTAZAR) study (*n* = 501), in which plasma Aβ_1− 42_ levels were 36.9 ± 11.7 pg/mL determined using ELISA INNOTEST^®^ [35]. Accordingly, some apparent gains in diagnostic performance may reflect denominator volatility in Aβ_1− 42_ rather than a disease-related signal in p-tau217.

Because the p-tau217/Aβ_1−42_ ratio is hyperbolically sensitive to the denominator, Aβ_1−42_ functions as the stability-limiting component. Even modest biological or analytical fluctuations in Aβ_1−42_ propagate inversely and disproportionately into the quotient. As a hypothetical numerical illustration, if p-tau217 is held constant, a 10% decrease in Aβ_1−42_ inflates the ratio by approximately 11%, whereas a 20% decrease inflates it by about 25%. Small absolute errors in Aβ_1−42_ values, which are more significant in the healthy population (due to lower values), can therefore produce large shifts in the ratio and may spuriously move values across diagnostic thresholds. This denominator-driven amplification suggests that some gains in diagnostic performance may reflect volatility in Aβ_1–42_ rather than true disease-related signal in p-tau217.

Misclassification is not only a matter of reduced power. In trial settings, it can lower the proportion of truly target-positive participants, flatten apparent treatment effects, distort biomarker-defined subgroup comparisons, and shift observed progression rates away from those expected in a correctly enriched cohort. A concrete implication of denominator instability is that ratio-driven threshold crossing may distort clinical trial enrollment and outcome interpretation, not merely reduce statistical power. Because the p-tau217/Aβ_1−42_ ratio is hyperbolically sensitive to the denominator, two individuals with identical p-tau217 values may receive different biomarker classifications solely because of variation in Aβ_1−42_. For example, if p-tau217 is held constant at 0.26 pg/mL, reported control-range Aβ_1−42_ values of 2.72–11.09 pg/mL [25] yield ratios from 0.023 to 0.096, a roughly fourfold spread. Likewise, with p-tau217 fixed at 1.45 pg/mL, the 95% confidence interval for plasma Aβ_1−42_ in AD patients in the BALTAZAR study (13.51–60.3 pg/mL) produces p-tau217/Aβ_1−42_ ratios spanning 0.024 to 0.107, representing more than a 4.4-fold variation [35]. If biomarker thresholds are used for trial enrichment, such denominator-driven shifts may move biologically similar individuals across eligibility cutoffs, thereby diluting treatment effects, altering observed progression rates, and complicating biomarker-defined subgroup analyses.

## The Challenge of Reliable Biomarkers in AD Diagnosis and Stratification

The pursuit of robust, clinically actionable biomarkers for AD remains a substantial scientific and regulatory challenge. Despite decades of effort, no single biomarker, nor any ratio of biomarkers, has shown consistent reliability across the full clinical spectrum of AD.

Unlike disorders with more direct biochemical confirmation, AD still lacks a definitive in vivo diagnostic test. Widely used markers such as CSF Aβ/tau profiles and amyloid PET improve probabilistic classification but remain technically and interpretively imperfect relative to postmortem pathology. This uncertainty affects not only diagnosis, but also treatment decisions, trial eligibility, interpretation of therapeutic outcomes, and the real risk of informing individuals that they have AD when they may not.

### Statistical considerations: AUC inflation, enrichment bias, and limits of diagnostic precision

Alongside the biological simplifications discussed above, the statistical strategies used to validate biomarkers such as p-tau217/Aβ_1−42_ also deserve careful scrutiny. A recent meta-analysis suggests that the apparently strong diagnostic performance of plasma p-tau217 should be interpreted cautiously. Although pooled accuracy estimates were favorable, the analysis documented marked inter-study variability across assays and biological reference standards, indicating that performance is strongly dependent on methodological and cohort-specific conditions rather than reflecting a stable analytical property. Importantly, approximately 90% of included studies were judged to be at high risk of bias because thresholds were optimized post hoc rather than prespecified or externally validated [33]. This observation has direct implications for the interpretation of p-tau217/Aβ42 ratio studies: if the underlying p-tau217 signal was frequently established under methodologically favorable conditions, then the cohorts used to validate the ratio should not be regarded as neutral test populations, but rather as selected settings in which performance may be accentuated. Accordingly, the area under the operating receiver operating characteristic curve (AUC) values in Table 2 may be best viewed as upper-range estimates obtained under favorable validation conditions, not as fully transportable measures of real-world diagnostic accuracy.

**Table 2:** Diagnostic Performance of p-tau217 and p-tau217/Aβ42 Ratio Across Validation Cohorts

Study Cohort	*p*-tau217 AUC	*p*-tau217/Aβ42 AUC	Reference Standard	Intermediate %	References
Clinic (*n* = 391)	0.94–0.96	0.963–0.966	Aβ PET + tau PET	10.6%	[36]
Community (*n* = 121)	0.93–0.95	0.963–0.966	Aβ PET + tau PET	16.5%	[36]
Real-World (*n* = 200)	0.92–0.94	0.94–0.96	Aβ PET	12–15%	[37, 38]
Research (*n* = 350)	0.95–0.97	0.96–0.97	Aβ PET	8–12%	[36]

Validation studies commonly preselect participants who already fall into relatively well-defined clinical or biomarker categories, thereby excluding diagnostically ambiguous individuals with mixed pathologies, atypical presentations, or competing comorbidities. Sensitivity and specificity are therefore often estimated in samples that are not representative of the broader populations in which these tests are ultimately intended to operate. When applied in real-world memory-clinic settings, where overlap between syndromes, medication effects, vascular contributions, and non-AD neurodegenerative processes are frequent, diagnostic performance often declines, a limitation that is not always emphasized in regulatory summaries [39]. As noted above, predictive value depends strongly on disease prevalence. Accordingly, performance estimates derived in enriched research cohorts cannot be transferred uncritically to lower-prevalence clinical settings, where the proportion of false positives may rise substantially even when sensitivity and specificity appear strong [40–42].

Because predictive performance depends strongly on disease prevalence, estimates derived in enriched research cohorts cannot be transferred uncritically to lower-prevalence clinical settings, where even apparently favorable sensitivity and specificity may still yield a substantial false-positive burden. In parallel, the relative scarcity of calibration analyses remains an important limitation [43]. A biomarker may discriminate well at the group level while still misestimating the individual probability of underlying pathology. Without calibration, even apparently strong discrimination can translate into poorly estimated individual risk and misplaced diagnostic confidence [44]. Taken together, these considerations suggest that reported performance metrics may overstate diagnostic certainty, particularly when derived from enriched cohorts, unsupported by external calibration, and interpreted without explicit attention to prevalence. If such biomarkers are then used to define eligibility in therapeutic trials, imperfect biological stratification could attenuate observed treatment effects and complicate efficacy interpretation.

### The circular trap: Mutual reinforcement of assay bias

It is important to acknowledge that the field has not remained static in the face of this problem. Meaningful efforts are underway to reduce circularity, including the development of neuropathology-anchored cohorts, longitudinal multimodal validation strategies, and cross-consortium harmonization initiatives designed to improve comparability across assays, centers, and diagnostic frameworks. These are important advances and should be recognized. They do not yet fully resolve the underlying issue, however, because individual-level neuropathological confirmation remains limited, longitudinal and multimodal datasets are not yet uniformly standardized across settings, and many validation pipelines still rely on partially interdependent biomarkers and clinical classifications as reference points.

Despite the advances, a persistent problem in contemporary AD research is the emergence of a circular validation loop in which one incompletely validated diagnostic tool is used to support another, creating an appearance of reliability without fully independent verification (Table 3). The issue becomes especially important when both parameters used to compute a ratio are themselves vulnerable to analytical bias or ratio-driven threshold crossing. Under those conditions, a strong correlation between the two methods does not necessarily indicate accuracy; it may instead reflect internal consistency within an imperfect framework. Biomarker reliability is then inferred not from comparison with clinical or neuropathological truth, but from agreement with another incompletely validated method. The result can be a self-reinforcing evidentiary structure in which internal coherence is mistaken for correctness.

**Table 3:** Clinical diagnostic accuracy and neuropathological discordance in AD

Characteristic	Value/Range	References
Sensitivity of clinical diagnosis	70.9%-87.3%	[45]
Specificity of clinical diagnosis	44.3%-70.8%	[45]
False positive rate	29.2%-55.7%	[45]
False negative rate	12.7%-29.1%	[45]
Primary misdiagnoses (non-AD dementia)	FTD, LBD, VaD, HS	[45, 46]
AD pathology in clinically non-AD dementia	39%	[45]

**Fig. 1:**
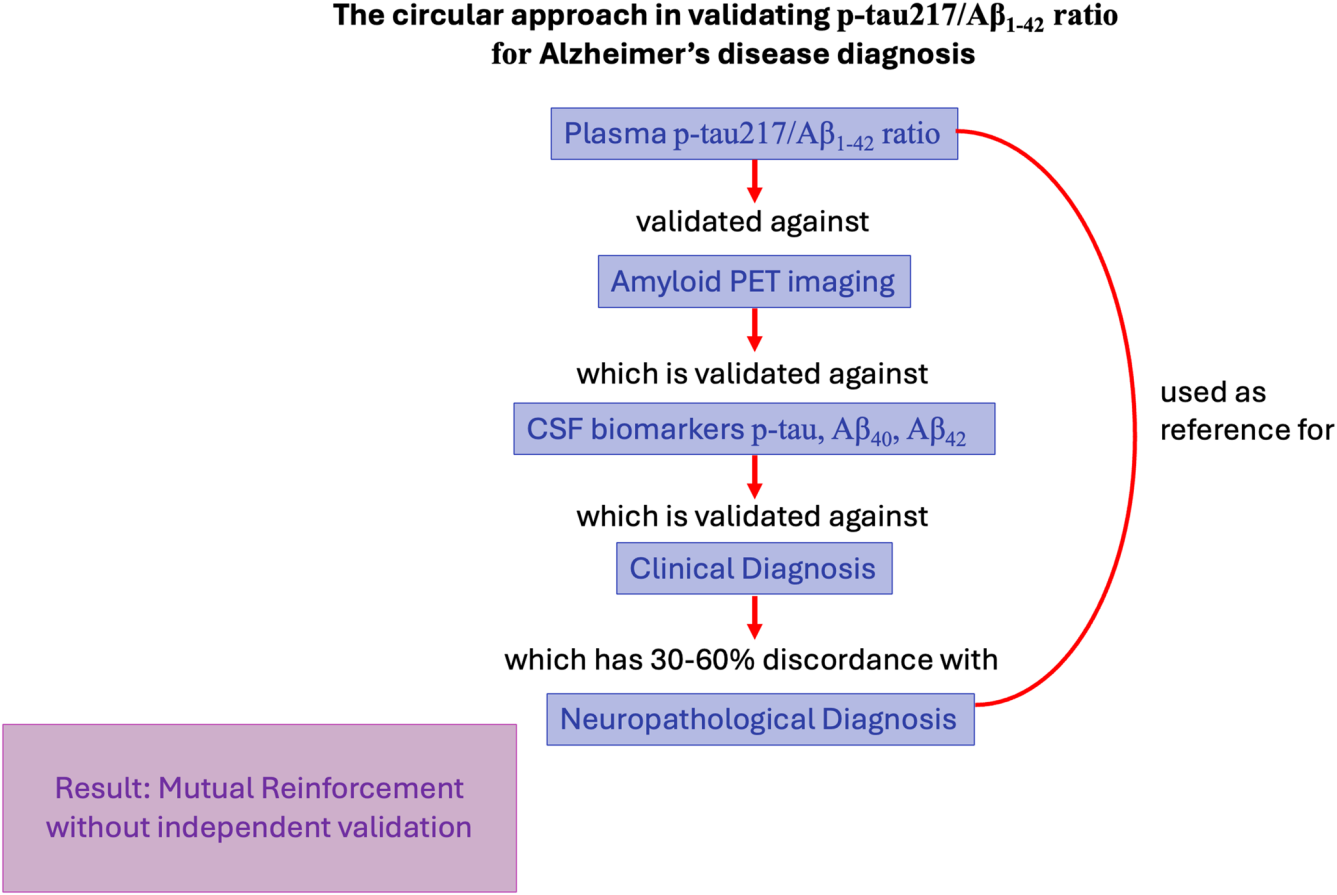
Schematic representation of the circular validation trap in AD biomarker development. Methods cross-validate one another in a self-reinforcing loop that may inflate apparent accuracy and propagate shared bias. CSF: Cerebrospinal fluid; PET: Positron emission tomography

Figure 1 illustrates this circular validation problem. Plasma p-tau217/Aβ42 is validated against amyloid PET, which is itself validated against CSF biomarkers (Aβ42, tau), which are validated against clinical diagnosis, even though clinical diagnosis shows 30–60% discordance with neuropathological diagnosis [46]. Neuropathology is then used post hoc to refine the imaging and CSF thresholds that subsequently serve as reference standards for plasma assays. The result is a self-reinforcing chain in which each imperfect method supports the next, while truly independent validation against individual-level neuropathology remains uncommon. A meta-analysis of clinicopathological correlation studies found that up to 30% of clinically diagnosed AD cases lacked sufficient neuropathological AD pathology at autopsy, whereas 20–30% of cognitively normal individuals showed substantial AD pathology. These findings complicate the assumption that biomarkers reflecting amyloid and tau necessarily predict clinical outcomes [45].

This concern is practical as well as conceptual. If diagnostic frameworks remain probabilistic and partially circular, a biomarker may appear accurate because it agrees with other imperfect tools rather than because it independently tracks disease truth. In that setting, treating a single biochemical ratio as a reference standard risks premature closure around an evidentiary structure that remains only partially validated.

Having outlined the analytical and biological limitations of the ratio, it is also important to indicate what kinds of approaches may offer a more cautious and constructive path forward.

## Possible analytical alternatives

If the p-tau217/Aβ_1−42_ ratio is analytically fragile and biologically difficult to interpret as a general normalization strategy, a constructive next step is to consider analytical approaches that preserve the distinct information carried by each biomarker. One reasonable alternative is to model p-tau217 and Aβ_1−42_ jointly as separate but potentially correlated variables, rather than combining them into a fixed quotient. Such an approach would allow their covariance structure to be represented explicitly while retaining their different biological meanings and avoiding some of the interpretive limitations introduced by ratio-based simplification.

In practice, modeling both biomarkers jointly as correlated variables with distinct variances and separate error structures may provide more faithful estimates of sensitivity, specificity, and overall accuracy than treating the quotient as if it were a single well-behaved variable. More concretely, a statistically appropriate alternative to the quotient would treat p-tau217 and Aβ_1−42_ as two separate but correlated predictors within the same model. At a minimum, this would involve a multivariable probabilistic framework, such as logistic or ordinal regression, in which each biomarker enters as its own term, with allowance for different error structures, possible nonlinearity, and an interaction term if justified empirically. More complete implementations could incorporate assay-specific calibration parameters, prevalence adjustment, and explicit measurement-error components, so that uncertainty in each analyte is propagated into the final predicted probability rather than hidden inside a single derived ratio. In longitudinal settings, mixed-effects or Bayesian latent-variable models could further distinguish within-person change from between-person heterogeneity.

A related consideration is that analytical and pre-analytical uncertainty may need to be incorporated more explicitly into future modeling efforts. This may be especially relevant if the measured p-tau217 signal reflects a heterogeneous composite of molecular species rather than a single well-defined entity. Under such conditions, multivariate or latent-variable frameworks may offer a more appropriate way to represent the observed data than a scalar ratio, since they can accommodate measurement uncertainty without obscuring the biological distinctness of the underlying components.

At the assay level, future progress may also benefit from approaches that move beyond reliance on a single epitope-dependent readout. Panel-based phospho-tau strategies, or other more molecularly resolved methods, may help distinguish among different p-tau217-containing species that are currently collapsed into a single signal. Where technically feasible, workflows such as immunoprecipitation followed by mass spectrometry could contribute to a more specific characterization of the molecular species being measured and, potentially, to a more quantitative interpretation of their relative abundance.

Accordingly, the successful validation of a platform-specific implementation should not be mistaken for the emergence of a method-independent gold standard; rather, it indicates that a given analytical system may be clinically useful within the boundaries in which it was derived and validated.

### Contexts in which the p-tau217/Aβ_1−42_ ratio may be defensible

Despite its limitations, the plasma p-tau217/Aβ_1−42_ ratio may still be defensible in restricted and carefully controlled settings, especially in symptomatic, preselected populations enriched for AD-type pathology and evaluated on a single harmonized platform using assay-specific cutoffs. Arguably, its strongest potential application is in within-individual longitudinal monitoring, where the interpretive burden is shifted away from universal thresholds and toward tracking directional change over time in the same person. Under such conditions, the ratio may also serve as a pragmatic tool for triage, cohort enrichment, or probabilistic prioritization for more definitive downstream testing. This, however, is a contextual and operational role, not evidence that the ratio constitutes a universally interpretable or mechanistically sufficient diagnostic standard. Limited value for serial follow-up should therefore not be conflated with robust cross-sectional diagnostic validity across populations, platforms, and biological contexts.

## Data Availability

Not applicable.
